# Similar aggregate but divergent disaggregate responses in human and AI under sequential reward-penalty incentives

**DOI:** 10.1016/j.isci.2026.117800

**Published:** 2026-10-06

**Authors:** Sihan Tao, Linghao Wang, Zheng Zhu, Der-Horng Lee

**Affiliations:** 1 Institute of Intelligent Transportation Systems & Polytechnic Institute, Zhejiang University, Hangzhou, China; 2 Institute of Intelligent Transportation Systems, College of Civil Engineering and Architecture, Zhejiang University, Hangzhou, China; 3 Zhejiang University–University of Illinois Urbana Champaign Institute, Zhejiang University, Hangzhou, China

**Keywords:** human-AI decision divergence, large language models, behavior simulation

## Abstract

With the rapid advancement of general-purpose AI, large language models (LLMs) are increasingly being used to simulate human decisions in structured environments. However, whether similarity in aggregate human and LLM responses extends to behavior under sequential reward-penalty incentives remains unclear. We address this question by introducing an established carbon mechanism to a repeated e-commerce delivery task and comparing decision responses between human, DeepSeek-v.3.2, and Qwen3-Max participants, wherein standard and slow delivery options are provided. In baseline settings, all participants predominantly select the theoretically dominant option. Under sequential reward-penalty incentives, all participant types shift toward slow delivery and exhibit comparably large aggregate changes. However, this aggregate-level similarity does not extend to “value of time” (VOT)-stratified response patterns and longitudinal trajectories. Humans exhibit gradual and incomplete adjustment, whereas LLMs display model-specific responses, including more stable or variable adjustment patterns depending on the model configuration. Thus, similar aggregate responses can coexist with divergent disaggregate behavioral patterns.

## Highlights

• Sequential reward-penalty incentives elicit similar aggregate human and AI responses
• Similar aggregate responses mask differences across value of time groups
• Humans adjust gradually, while tested LLMs show distinct temporal patterns
• Aggregate agreement alone cannot validate LLMs as human behavioral proxies

## Graphical abstract

**Figure:**
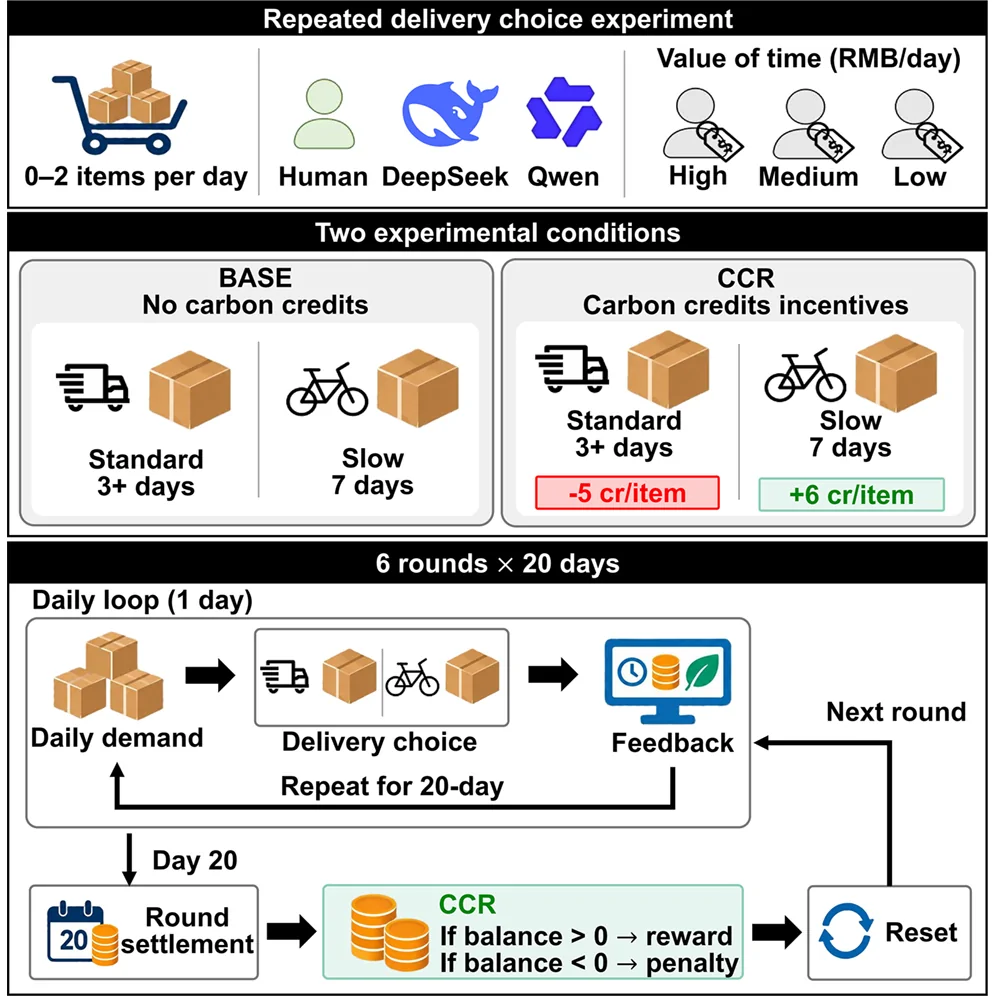

## Teaser

Applied sciences; Artificial intelligence

## Introduction

### Contexts and literature review

Deep learning has progressed from task-specific perceptual models toward foundation models with broader capabilities. Advances in visual perception demonstrate increasingly sophisticated feature representation, semantic and contextual modeling, and robustness in complex environments.^1^^,^^2^^,^^3^^,^^4^^,^^5^ Within this broader technological trajectory, large language models (LLMs) are increasingly being explored beyond text generation and knowledge-based question answering for reasoning and behavioral simulation.^6^^,^^7^^,^^8^ In the fields of engineering, management science, social science, etc., a growing number of scholars are adopting LLMs as a novel behavioral simulation tool, constructing generative agents equipped with role settings and memory mechanisms to model individual decision-making in virtual environments.^9^ This approach offers a scalable experimental paradigm for policy simulation and social system research, helping to address the limitations of traditional human experiments in terms of large sample sizes, high cost, and reproducibility.^10^

Following this background, existing research has expanded from classical game theory paradigms^11^^,^^12^^,^^13^ to more complex real-world decision contexts, such as financial investment decisions,^14^ daily travel choices,^15^ and social reasoning games.^16^^,^^17^ Some studies indicate that in well-structured tasks with clear rules, LLMs can effectively replicate human behavioral patterns.^18^ However, other research has found the biases generated by LLMs when tasks involve complex contextual reasoning or require prediction of real human behavior.^19^^,^^20^ More recent research has further argued and demonstrated that similarities in human and LLM outputs can conceal substantial differences in the judgment strategies, epistemic structures, and cognitive processes that produce them.^21^^,^^22^^,^^23^^,^^24^

However, an important but comparatively underexplored question is whether similarity in aggregate human and LLM responses extends to behavior under sequential reward-penalty incentives. Unlike conventional one-shot experimental settings, real-world incentive policies often involve cumulative incentives, delayed feedback, and repeated choices that require behavioral adjustment over time. Clarifying this question is important for determining whether LLM-based simulations capture only aggregate policy responses or also the disaggregate behavioral patterns through which those responses emerge.

To bridge this gap, this study employs a reward-penalty carbon credit mechanism in e-commerce logistics as a testbed. With the rapid growth of online retail,^25^ last-mile delivery has become a major contributor to urban carbon emissions.^26^^,^^27^ Some studies have begun exploring demand-side incentive mechanisms to encourage consumer participation in low-carbon transitions.^28^^,^^29^^,^^30^ While existing platforms (e.g., Amazon^31^) use one-shot incentives such as vouchers to encourage greener delivery options, these mechanisms often lack sustained behavioral guidance. By contrast, the credit-charge-cum-reward (CCR)^32^^,^^33^ mechanism introduces cumulative reward/penalty incentives and periodic settlement, requiring users to balance delivery speed (a standard option) against long-term carbon credit (a slow option) outcomes across sequential multi-day decisions.

Building on this framework, we combine human experiments with LLM simulations (DeepSeek-v.3.2 and Qwen3-Max) to construct a multi-day repeated decision environment. Baseline experiments (i.e., no CCR) serve as an implementation benchmark by testing whether humans and LLMs predominantly select the theoretically dominant option. The CCR mechanism is then introduced as a sequential reward-penalty incentive environment with repeated decisions and intertemporal trade-offs. We compare human, DeepSeek-v.3.2, and Qwen3-Max participants across three dimensions: policy responses, longitudinal response trajectories, and exploratory strategy portraits. While all participants show comparably large aggregate responses to CCR under the tested experimental composition, this aggregate-level similarity does not extend to “value of time” (VOT)-stratified response patterns and longitudinal trajectories. Humans exhibit gradual and incomplete adjustment, whereas LLMs display model-specific disaggregate behavioral patterns characterized by distinct temporal trajectories and decision-profile compositions despite similar aggregate responses. These findings indicate that similar aggregate responses can coexist with divergent disaggregate behavioral patterns, limiting the use of aggregate correspondence alone to validate LLMs as proxies for sequential reward-penalty policy response simulations.

### Baseline experiment

The baseline experiment (labeled BASE later) simulates a traditional e-commerce environment without incentives or constraints; participants decide on delivery options based solely on their inherent preferences for delivery speed. To quantify these preferences, we introduce the concept of VOT from transportation engineering into the e-commerce logistics context.^34^^,^^35^ VOT originally refers to the monetary amount a traveler is willing to pay to save unit travel time. In this study, VOT characterizes the monetary cost a consumer is willing to pay to save unit delivery time. In each experiment group, 12 participants are assigned to three equal-size groups corresponding to preset VOT values of high (18 RMB/day), medium (10 RMB/day), and low (3 RMB/day).

The experiment consists of six independent rounds, each representing a complete 20-day settlement period. On each virtual decision day, the system generates daily purchase demands (0, 1, or 2 items) for each participant. This design aims to reflect real-life scenarios where consumer demand is not evenly distributed but exhibits stochasticity and fluctuations. Participants must decide the number of items to be delivered via standard delivery, with any remaining items automatically allocated to slow delivery. For experimental tractability, the two delivery options are modeled as stylized proxies that differ in both delivery speed and relative carbon intensity. Standard delivery is modeled as the relatively higher-emission option, reflecting the possibility that tighter fulfillment requirements can reduce opportunities for shipment consolidation and load-factor optimization. Slow delivery is modeled as the relatively lower-emission option because the longer fulfillment window may provide greater flexibility for consolidation and routing optimization. These labels represent an experimental abstraction rather than a universal characterization of real-world delivery services. Actual logistics emissions depend on multiple operational factors, including routing, vehicle and energy type, warehouse location, delivery density, and product characteristics. Specifically, the base delivery time for standard delivery is 3 days. To simulate logistics pressure, the system incorporates a non-linear delay feature where higher demand leads to longer delivery times. In contrast, slow delivery is fixed at 7 days. Additionally, to simulate the existing delivery situation of major platforms such as JD.com and Amazon, the delivery costs for both delivery methods are set to 0 RMB. Therefore, the daily delivery cost for each participant in BASE equals to his/her VOT multiplied by the delivery time.

Given the decisions made by the participants, the system computes the delivery time and provides daily feedback (Figure 1B), including contextual information (e.g., total rounds, current round, current day, assigned VOT group, number of items bought today, last standard delivery time, and slow delivery time) and performance feedback (e.g., yesterday’s delivery cost and the current round’s cumulative cost). With such feedback, the participants are expected to dynamically refine their decisions for the next day. Furthermore, a summary of the cumulative cost is provided at the conclusion of each 20-day round, providing a holistic performance evaluation. Participants’ monetary experiment rewards are positively correlated with the total costs saved, which governs their sequential day-to-day decisions.

**Figure 1:**
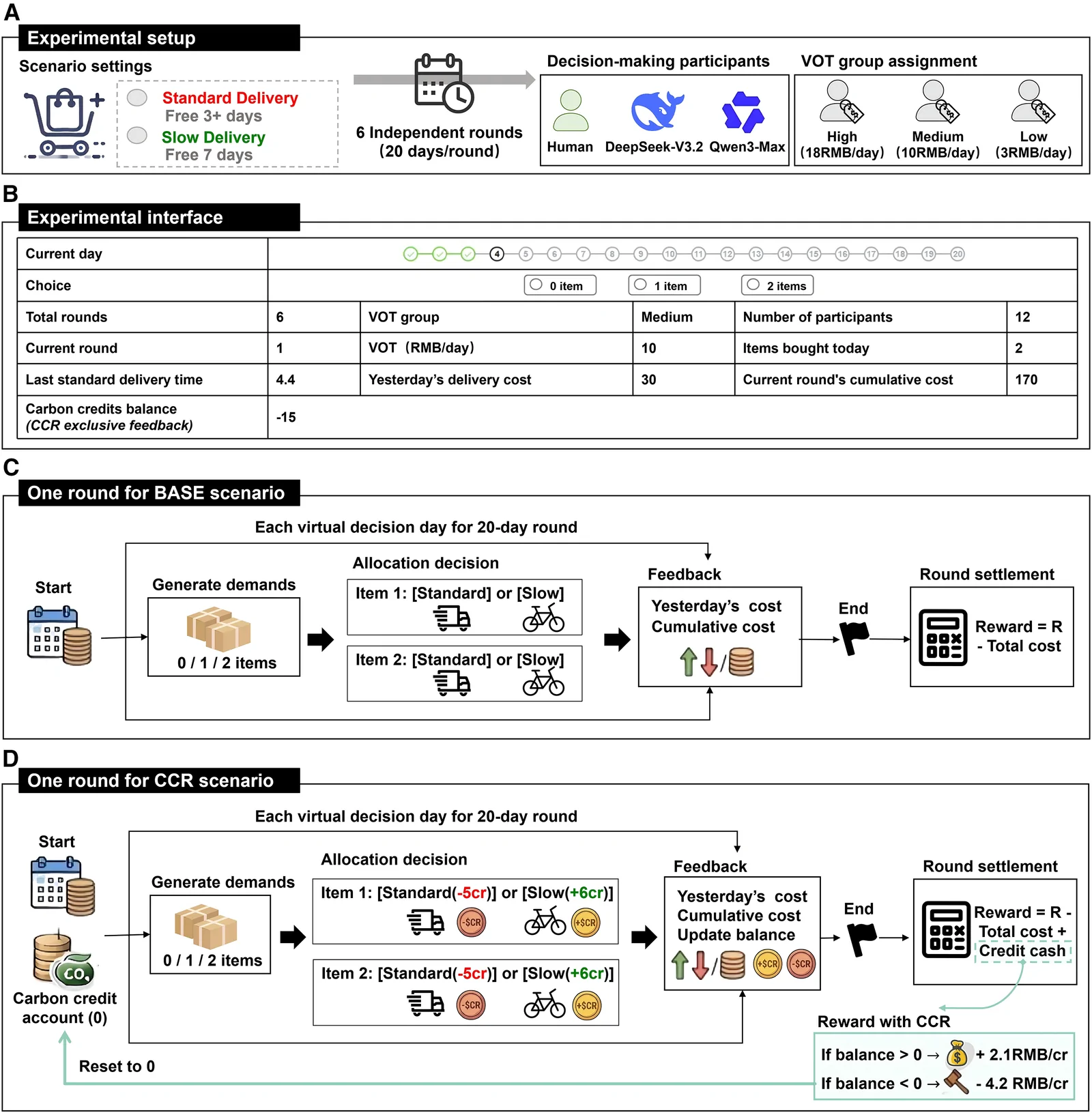
Experimental framework for e-commerce delivery choice analysis (A) Experimental setup. (B) Experimental interface. (C) One round for BASE scenario. (D) One round for CCR scenario.

We conduct 5 groups of human experiments (i.e., all participants are humans) and 10 groups of LLM experiments (i.e., all participants are LLM agents, with 5 groups of DeepSeek-v.3.2 and 5 groups of Qwen3-Max) for comparison. In the LLM experiments, the same task logic, parameter settings, and daily feedback are fed to DeepSeek-v.3.2 and Qwen3-Max through well-designed structured prompts, ensuring that humans and LLMs operate under complete informational symmetry.

BASE serves as an implementation check for both humans and LLMs. Before examining responses under sequential reward-penalty incentives, we first verify whether the experimental implementation produces the theoretically expected choice under a transparent incentive structure. The following research question and hypothesis are proposed:

Research question 1: Do humans and LLMs predominantly select the theoretically dominant delivery option in BASE?

Hypothesis 1 (H1): Humans and LLMs will predominantly select standard delivery because it is the theoretically dominant option across all VOT groups in BASE.

Although standard delivery may experience demand-related delays, its delivery time remains shorter than the fixed lead time of slow delivery (see STAR Methods: validation of statistical analysis). Therefore, the standard delivery is a rational choice for participants in all VOT groups. In BASE, the incentive structure is simple and transparent, requiring participants only to compare delivery times between the two options. Given this limited strategic complexity, humans and LLMs are, therefore, expected to exhibit similar aggregate speed-first choices. To validate H1, we conduct two specific comparisons: (1) the deviation between participants’ actual standard delivery proportions and the theoretical equilibrium; and (2) differences in standard delivery proportions among participants. The standard delivery proportion is calculated based on the last two of the six-round experiments to capture stable behavioral patterns.

Preliminary experimental results support H1, as humans and LLMs exhibit similar aggregate choice patterns in BASE, with all participant types predominantly selecting standard delivery, the theoretically dominant option. While BASE confirmed that both humans and LLMs respond appropriately to a transparent dominant option, it also raises a deeper research question: whether humans and LLMs exhibit similar aggregate outcomes and response patterns in a sequential reward-penalty incentive environment that involves repeated decisions and intertemporal trade-offs? To explore this, the CCR mechanism and the corresponding experiment are introduced on the basis of BASE.

### CCR experiment

Under the CCR mechanism (Figure 1D), each participant is assigned an independent carbon credit account with an initial balance of zero, operating on a 20-day settlement round. On each virtual decision day, the participants make trade-offs between the delivery time preferences and the credit gains/losses based on their delivery decisions. Specifically, each standard delivery of one item deducts 5 credits, while each slow delivery selection rewards 6 credits, with balances allowed to be negative. At the end of one round, if the account balance is positive, credits are converted into cash rewards at a rate of 2.1 RMB per credit; if negative, a penalty is charged at 4.2 RMB per credit. After settlement, the carbon credit account is reset to zero for the subsequent round. The CCR experimental interface provides daily carbon credits balance and specific reward/penalty parameters. This allows the participants to track their credit status and anticipate settlement outcomes at the end of the round.

The CCR experiment is designed to examine how humans and LLMs respond when confronted with a sequential reward-penalty incentive environment involving repeated decisions and intertemporal incentive trade-offs. Under the CCR experiment, this study proposes three dimensions of scientific questions:

Question 2: How do human and LLM decision behaviors respond to the CCR mechanism?

Hypothesis 2.1 (H2.1): The CCR mechanism will significantly increase the adoption of slow delivery among both humans and LLMs.

Hypothesis 2.2 (H2.2): Despite potentially similar aggregate CCR effects, humans and LLMs will exhibit different VOT-stratified policy response patterns.

Under the CCR mechanism, choosing slow delivery allows participants to accumulate carbon credits, while excessive reliance on standard delivery may lead to penalties at settlement. Therefore, both humans and LLMs are expected to increase the adoption of slow delivery compared with BASE. However, similar aggregate responses need not imply identical policy response patterns across VOT groups. Human decision-making is often shaped by bounded rationality,^36^^,^^37^^,^^38^^,^^39^ where individuals may rely on heuristics and exhibit systematic deviations from incentive-optimal behavior. In contrast, LLMs may more directly map decision options to the explicitly defined rules embedded in the mechanism, potentially producing choices that more consistently follow the designed incentive logic, although the extent of this consistency may vary across models. Empirically, we examine the standard delivery proportion to measure the behavioral shift induced by CCR. To examine VOT-stratified policy response patterns, we further analyze the deviation from the theoretical benchmark and differences in delivery choices within each VOT group.

Question 3: How do the longitudinal response trajectories of humans and LLMs differ when responding to the CCR mechanism?

Hypothesis 3 (H3): Humans are expected to exhibit gradual and potentially incomplete adjustment toward the theoretical benchmark, whereas LLMs are expected to display model-specific temporal response patterns.

Human learning processes often rely on trial-and-error feedback and the accumulation of experience and may, therefore, be characterized by early fluctuations followed by gradual convergence.^40^ In contrast, rather than depending primarily on experience accumulation, LLM responses are generated without parameter updating and are conditioned on the policy rules, current state information, and recent interaction history provided in the prompt. Changes in LLM choices across rounds may, therefore, reflect in-context conditioning, repeated evaluation of the available information, and stochastic generation, which may lead to faster convergence or qualitatively different adjustment paths. To evaluate H3, we examine round-level trajectories of standard delivery proportions and best response rates (BRRs), convergence toward the corresponding theoretical benchmarks, and conditional choice persistence across purchase days.

Question 4: What VOT-stratified strategy portraits are observed for human participants, DeepSeek-v.3.2, and Qwen3-Max under the CCR mechanism?

Question 4 is addressed through an exploratory descriptive analysis. Aggregate correspondence does not necessarily indicate that delivery choices are organized similarly across participant types and VOT groups. We, therefore, use exploratory clustering to characterize the observable allocation-frequency patterns under CCR. A strategy mode is defined as a descriptive category of choice frequencies across one-item and two-item purchase scenarios, whereas a strategy portrait represents the composition of these modes within each participant type and VOT group. Because the human sample comprises different individuals, whereas each LLM group comprises repeated generations from a fixed configuration, this analysis documents the strategy-mode compositions observed under the specific configurations tested.

## Results

Theoretical simulations of the equilibrium states under BASE and CCR were conducted to establish a benchmark for subsequent analysis (see STAR Methods: theoretical equilibrium). The theoretical simulation results in BASE (Table 1) show a 100% standard delivery proportion, regardless of consumer VOT heterogeneity. With the introduction of CCR, the proportion of standard delivery for the system decreases to 51.39%, exhibiting distinct stratified response characteristics across different VOT levels.

**Table 1:** Theoretical simulation equilibrium results

Scenarios	Overall	High VOT	Medium VOT	Low VOT
BASE	100.00%	100.00%	100.00%	100.00%
CCR	51.39%	100.00%	54.17%	0.00%

### Baseline implementation check (question 1)

In BASE, the performance of humans, DeepSeek-v.3.2, and Qwen3-Max was compared in terms of theoretical deviation and difference among participant types.

First, by examining the degree of deviation from the theoretical simulation, we verified whether each participant type adhered to the speed-first rational expectation. The observed proportions of standard delivery were 0.989 for humans, 0.997 for DeepSeek-v.3.2, and 0.981 for Qwen3-Max. These proportions deviated from the theoretical benchmark by −1.14, −0.31, and −1.85 percentage points, respectively. We quantified uncertainty using a system-level bootstrap that resamples independent systems. The resulting 95% confidence intervals (CIs) were (0.982, 0.994) for humans, (0.994, 0.999) for DeepSeek-v.3.2, and (0.944, 1.000) for Qwen3-Max. Together, these results confirmed that the experimental implementation elicited the theoretically dominant speed-first choice across all participant types.

Second, we examined whether the decision performance differed across participants. We fitted a binomial generalized estimating equation with system as the highest-level clustering unit and calculated bias-reduced cluster-robust standard errors. The overall participant-type effect was not statistically significant (Wald *χ*^2^(2) = 4.777, *p* = 0.092). For DeepSeek-v.3.2 relative to humans, the estimated adjusted risk difference was +0.81 percentage points (95% CI: [0.04, 1.90]; *p*_Holm_ = 0.081). For Qwen3-Max relative to humans, the corresponding estimate was −0.77 percentage points (95% CI: [−14.08, 1.55]; *p*_Holm_ = 0.653). Thus, after correction for multiple comparisons, we found no evidence that either LLM differed from humans in selecting the dominant BASE option.

We further aggregated choices within each independent system and compared participant types separately within each VOT group. Exact system-label permutation Kruskal-Wallis tests identified no multiplicity-adjusted differences in the high-VOT group (*H* = 4.304, *p*_Holm_ = 0.333). The same conclusion applied to the medium-VOT group (*H* = 1.550, *p*_Holm_ = 0.882) and low-VOT group (*H* = 0.692, *p*_Holm_ = 0.882).

Taken together, the BASE results confirm task implementation and the predominant selection of a simple dominant option, providing support for H1.

### The effectiveness of CCR and human-AI response patterns (question 2)

Having confirmed correct task implementation in BASE, we next examined how humans and LLMs responded to the sequential reward-penalty incentives under CCR. The analysis evaluated the aggregate CCR effect, compatibility with the theoretical benchmarks, and VOT-stratified response patterns.

First, the experimental results confirmed that the CCR mechanism exerted a significant influence on delivery preferences, markedly reducing the proportion of standard delivery choices across all participant types (Figure 2A). The raw proportions decreased from 0.989 to 0.463 for humans, from 0.997 to 0.513 for DeepSeek-v.3.2, and from 0.981 to 0.450 for Qwen3-Max. In the system-clustered binomial generalized estimating equation (GEE), the adjusted CCR-BASE risk differences were −51.14 percentage points for humans (95% CI: [−60.82, −42.76]), −48.40 percentage points for DeepSeek-v.3.2 (95% CI: [−50.94, −45.72]), and −53.11 percentage points for Qwen3-Max (95% CI: [−61.37, −40.25]). All three effects remained significant after Holm correction (all *p*_Holm_ < 0.001) and in exact system-label permutation analyses (all *p*_Holm_ = 0.024). The participant × condition interaction was not significant (Wald *χ*^2^(2) = 2.172, *p* = 0.338), providing no evidence that the magnitude of the CCR effect differed among the three participant groups. Within CCR, the adjusted standard delivery probability differences ranged from −2.78 to +6.25 percentage points across the three pairwise participant-type contrasts; all 95% CIs included zero, and none remained statistically detectable after Holm correction (all *p*_Holm_ ≥ 0.280). These results demonstrate the effectiveness of the CCR mechanism on guiding participants toward slow delivery options, which supports H2.1.

**Figure 2:**
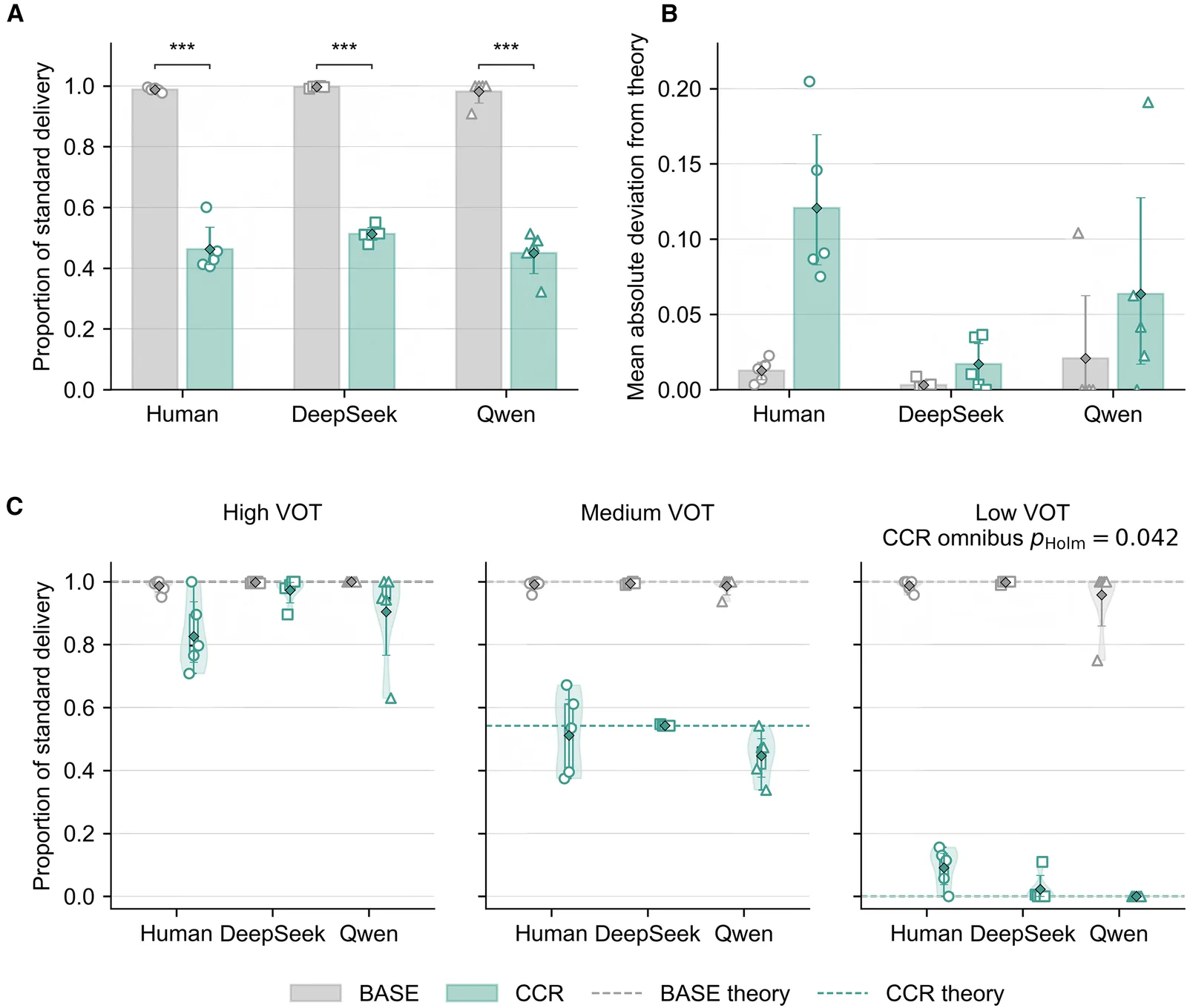
Behavioral responses under BASE and CCR (A) Comparison of standard delivery proportion. Bars represent pooled proportions, open symbols represent complete experimental systems, and diamonds with error bars denote pooled estimates and 95% system-cluster bootstrap confidence intervals. Asterisks indicate the BASE-CCR contrasts from the system-clustered binomial GEE after Holm correction. ∗∗∗*p*_Holm_ < 0.001. Standard delivery proportions were calculated as the number of purchased items allocated to standard delivery divided by the total number of purchased items. (B) Absolute deviation from theoretical benchmark. Bars show the mean absolute deviations from the theoretical benchmark across systems, open symbols show individual systems, and diamonds with error bars denote means and 95% system-bootstrap confidence intervals. (C) Distribution of standard delivery proportions among different VOT groups. Violin envelopes show descriptive system-level distributions; narrow boxes show the interquartile range and median; open symbols show individual systems; and diamonds with error bars denote pooled estimates and 95% system-cluster bootstrap confidence intervals. Gray and green dashed lines indicate the BASE and CCR theoretical benchmarks, respectively. The BASE benchmark was 1.000 for all VOT groups, whereas the CCR benchmarks were 1.000, 0.542, and 0.000 for high, medium, and low VOT. The displayed low-VOT omnibus result was obtained using a system-level exact Kruskal-Wallis test with Holm correction across the three VOT groups. Circles, squares, and triangles represent the human, DeepSeek-v.3.2, and Qwen3-Max systems, respectively. The complete experimental system was the independent unit (*n* = 5 systems per participant type and condition).

Second, we compared the observed aggregate proportions with the theoretical benchmark implied by CCR. The observed proportions were 0.463 for humans, 0.513 for DeepSeek-v.3.2, and 0.450 for Qwen3-Max, corresponding to deviations of −5.11, −0.11, and −6.36 percentage points. System-clustered GEE analyses yielded 95% CIs of (−13.43, 3.42), (−2.58, 2.37), and (−13.54, 1.04), respectively. None was statistically detectable after Holm correction. Thus, under the tested VOT composition, the pooled aggregate estimates were compatible with the theoretical benchmark within the available uncertainty.

The VOT-stratified analysis revealed differences that were obscured by aggregate outcomes. For humans, the observed standard delivery proportions were 0.826, 0.512, and 0.092 in the high-, medium-, and low-VOT groups, respectively. The corresponding proportions were 0.973, 0.543, and 0.023 for DeepSeek-v.3.2 and 0.904, 0.447, and 0.000 for Qwen3-Max. Because the theoretical benchmarks for the high- and low-VOT groups were the boundary values of 1 and 0, respectively, these strata were summarized using deviations and system-cluster bootstrap intervals. At high VOT, DeepSeek-v.3.2 was numerically closest to the theoretical benchmark, whereas humans showed the largest pooled deviation. At medium VOT, neither humans nor DeepSeek-v.3.2 differed detectably from the theoretical value of 0.542. Qwen3-Max was 9.48 percentage points below the benchmark. This deviation was detected in the system-clustered GEE (*p*_Holm_ = 0.044) but not in the exact five-system sign-flip sensitivity analysis (*p*_Holm_ = 0.188). It was, therefore, interpreted as suggestive rather than robust evidence of deviation. At low VOT, humans retained residual standard delivery choices (0.092; 95% CI: [0.043, 0.138]), whereas DeepSeek-v.3.2 produced a smaller proportion of 0.023, and all Qwen3-Max systems reached zero.

System-level exact Kruskal-Wallis tests detected no overall participant difference in the high-VOT group (*H* = 2.810, *p*_Holm_ = 0.299) or medium-VOT group (*H* = 3.803, *p*_Holm_ = 0.299). An overall participant difference was detected in the low-VOT group (*H* = 7.102, *p*_Holm_ = 0.042). However, no individual pairwise contrast remained significant after family-wide Holm correction. The low-VOT result, therefore, indicates an overall distributional difference.

Figure 2C further shows that CCR changed not only average the choice levels but also the pattern of between-system dispersion. Under BASE, system-level standard delivery proportions remained close to the ceiling across nearly all participant types and VOT combinations, leaving limited variation in most strata. Under CCR, human systems retained appreciable heterogeneity across all three VOT strata: system-level proportions ranged from 0.708 to 1.000 for high VOT, from 0.375 to 0.672 for medium VOT, and from 0.000 to 0.156 for low VOT. DeepSeek-v.3.2 was markedly more concentrated, particularly in the medium-VOT group, where all five systems fell between 0.542 and 0.547, close to the interior theoretical benchmark. Qwen3-Max showed a different dispersion profile, with system-level proportions ranging from 0.630 to 1.000 for high VOT and from 0.339 to 0.542 for medium VOT, followed by complete convergence to zero standard delivery choices in all five low-VOT systems. Human behavior showed the most persistent between-system heterogeneity across the VOT groups, whereas variability in the two LLMs was more localized and alternated with highly concentrated or boundary-convergent responses.

Taken together, CCR produced a large and consistent aggregate shift away from standard delivery across humans and both LLMs. The magnitude of this aggregate effect did not differ detectably among the three groups, and their aggregate outcomes were compatible with the theoretical benchmark under the tested composition. The VOT-stratified results, nevertheless, identified distinct descriptive patterns. Humans retained residual standard delivery choices at low VOT and persistent heterogeneity across the VOT groups. DeepSeek-v.3.2 produced highly concentrated outcomes close to the theoretical benchmark. Qwen3-Max showed a suggestive downward deviation at medium VOT and boundary convergence at low VOT. These results provide support for H2.2.

### System convergence process (question 3)

By tracking the longitudinal response trajectories over 120 decision days across six settlement rounds, we examined observable response dynamics when encountering CCR policy incentives. VOT-stratified longitudinal models analyzed standard delivery choices, with the complete experimental system as the clustering unit. The analyses evaluated round-level choice trajectories, round-level BRR, convergence toward the theoretical benchmark, and conditional choice persistence (Figure 3).

**Figure 3:**
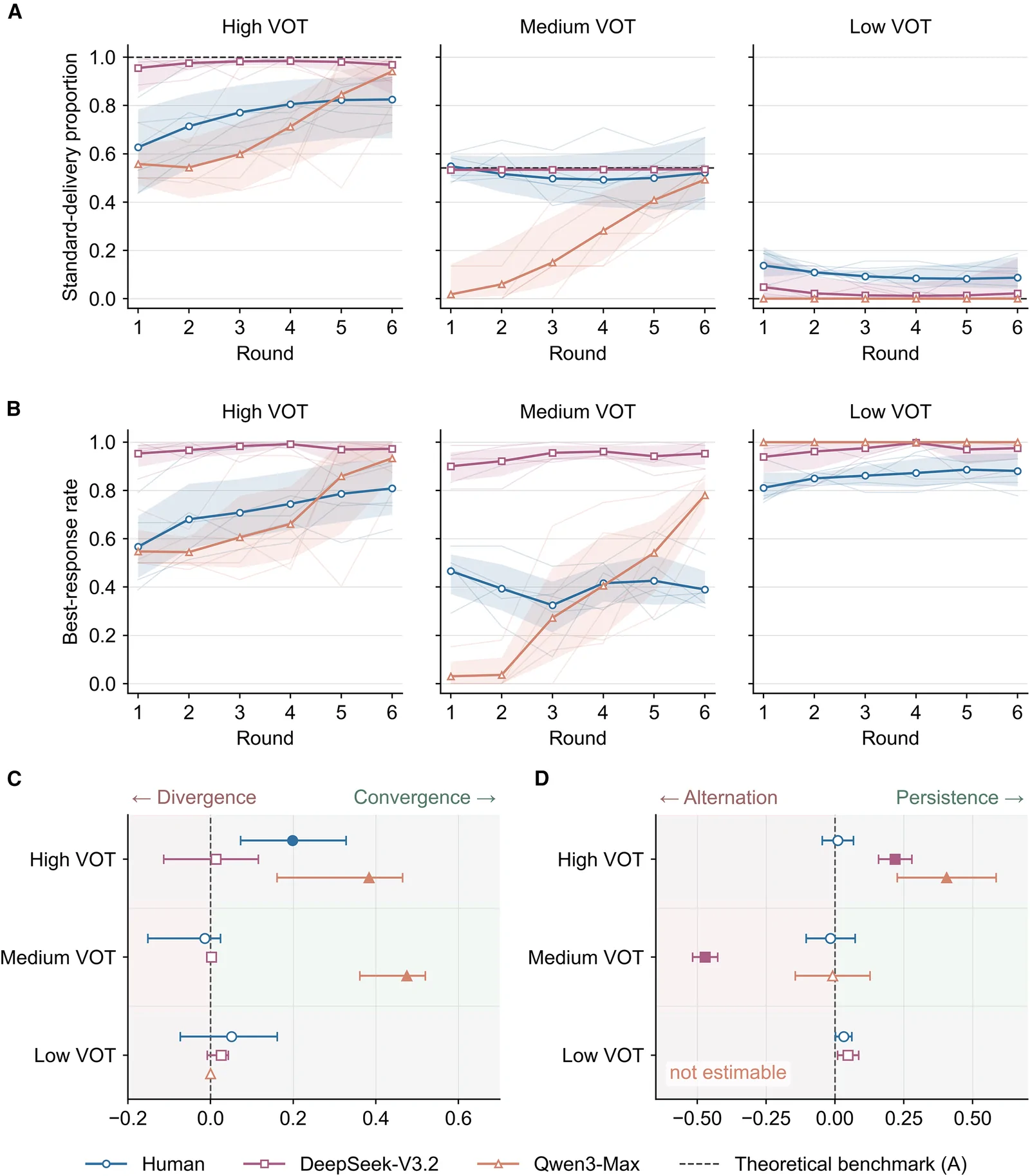
Dynamic results of experiments under CCR (A) The 6-round trajectories of standard delivery proportion. Thick lines show participant-type means, thin lines represent individual systems, and shaded bands denote 95% confidence intervals. Dashed horizontal lines indicate the corresponding theoretical benchmarks (1.000, 0.542, and 0.000). (B) The 6-round trajectories of best response rate. Thick lines show participant-type means, thin lines represent individual systems, and shaded bands denote 95% confidence intervals. (C) Convergence toward the theoretical benchmark. Points represent the absolute deviation from the theoretical benchmark in round 1 minus that in round 6. Horizontal intervals denote 95% confidence intervals, and positive values indicate movement toward the benchmark. Two-sided simulation-based *p* values were calculated from the corresponding distribution of convergence estimates. Filled symbols indicate *p*_Holm_ < 0.05, whereas open symbols indicate *p*_Holm_ ≥ 0.05. (D) Conditional choice persistence. Adjusted difference in the probability of choosing standard delivery for the current item, comparing cases in which all versus none of the items were assigned to standard delivery on the previous purchase day. Symbols denote point estimates, and horizontal intervals denote 95% confidence intervals. Statistical significance was assessed using two-sided Wald tests. Filled symbols indicate *p*_Holm_ < 0.05, whereas open symbols indicate *p*_Holm_ ≥ 0.05. Qwen3-Max in the low-VOT group remained at the zero boundary, and its conditional lag effect was, therefore, not estimable. Multiplicity was controlled using the Holm procedure within prespecified test families. The complete experimental system was the independent unit (*n* = 5 systems per participant type).

Round-level trajectories differed among participants in the medium-VOT group (Wald *χ*^2^(4) = 18.625, *p*_Holm_ = 0.003). The Qwen3-Max trajectory differed from the human (*p*_Holm_ = 0.009) and DeepSeek-v.3.2 trajectories (*p*_Holm_ = 0.010), whereas the latter two showed no detectable difference (*p*_Holm_ = 0.315). For Qwen3-Max, the adjusted standard delivery probability increased from 0.018 in round 1 to 0.493 in round 6. Its absolute deviation from the theoretical benchmark decreased by 0.475 (95% CI: [0.361, 0.520]; *p*_Holm_ = 0.003). This reduction exceeded those observed for humans and DeepSeek-v.3.2 (both *p*_Holm_ < 0.003). At high VOT, the corresponding probabilities increased from 0.627 to 0.825 for humans and from 0.558 to 0.941 for Qwen3-Max. Their absolute deviations decreased by 0.198 (95% CI: [0.072, 0.328]; *p*_Holm_ = 0.017) and 0.383 (95% CI: [0.160, 0.464]; *p*_Holm_ = 0.045), respectively. DeepSeek-v.3.2 remained close to the theoretical benchmark, with adjusted probabilities of 0.956 and 0.969 in rounds 1 and 6, respectively. Participant-type-by-round interactions were not detectable at high or low VOT (both *p*_Holm_ = 0.094).

To complement the convergence analysis, we calculated a BRR (see STAR Methods: calculation of best response rate) within each 20-day settlement round (Figure 3B). An observed allocation was classified as a best response when it minimized one-step generalized cost given current demand, the carbon credit balance, the latest observed standard delivery time, and the terminal settlement rule. BRR, therefore, measures short-horizon response consistency under available information. Overall BRR was 0.666 for humans (95% CI: [0.636, 0.698]), 0.960 for DeepSeek-v.3.2 (95% CI: [0.948, 0.972]), and 0.679 for Qwen3-Max (95% CI: [0.631, 0.724]). The six-round BRR trajectories differed among participant types (participant-type-by-round interaction: Wald *χ*^2^(2) = 15.885, *p* < 0.001). Human BRR increased significantly at high VOT (odds ratio [OR] per round = 1.247, 95% CI: [1.061, 1.466]; *p*_Holm_ = 0.045) but showed no adjusted trend at medium or low VOT. DeepSeek-v.3.2 maintained consistently high BRR and showed a modest increase at medium VOT (OR = 1.175, 95% CI: [1.107, 1.247]; *p*_Holm_ < 0.001). Qwen3-Max BRR increased at both high VOT (OR = 1.534, 95% CI: [1.219, 1.930]; *p*_Holm_ = 0.002) and medium VOT (OR = 2.477, 95% CI: [1.776, 3.454]; *p*_Holm_ < 0.001). Its low-VOT BRR remained at 1.000 throughout, precluding slope estimation.

Conditional choice persistence differed among participants in the high- and medium-VOT groups (both omnibus *p*_Holm_ < 0.001). In the high-VOT group, the previous purchase day standard share corresponded to an adjusted probability increase of 0.218 for DeepSeek-v.3.2 (95% CI: [0.158, 0.279]; *p*_Holm_ < 0.001). The corresponding increase was 0.405 for Qwen3-Max (95% CI: [0.226, 0.585]; *p*_Holm_ < 0.001). The human estimate was 0.010 (95% CI: [−0.046, 0.067]; *p*_Holm_ = 1.000). In the medium-VOT group, DeepSeek-v.3.2 showed a negative adjusted probability difference of −0.471 (95% CI: [−0.517, −0.426]; *p*_Holm_ < 0.001). The human and Qwen3-Max estimates were −0.016 (95% CI: [−0.105, 0.073]) and −0.009 (95% CI: [−0.145, 0.127]), respectively. Qwen3-Max remained at the zero boundary in the low-VOT group, and its conditional lag effect was not estimable.

These results were consistent with H3 and revealed systematic differences in longitudinal response trajectories across participants. Humans showed gradual and sometimes incomplete convergence, with modest BRR improvements concentrated in the high-VOT group. DeepSeek-v.3.2 maintained benchmark-proximate choices and high BRR, alongside VOT-dependent persistence or alternation. Qwen3-Max showed delayed cross-round adjustment and pronounced BRR increases at high and medium VOT. Thus, similar later-stage aggregate outcomes coexisted with distinct temporal response structures.

### Exploratory strategy portraits (question 4)

We performed exploratory unsupervised clustering based on delivery choice patterns for 1-item and 2-item orders (Figure 4). The features comprised the standard delivery proportion on 1-item days and the all-standard, mixed, and all-slow proportions on 2-item days. BASE and CCR observations were fitted jointly to define a common profile space, while the following comparisons focus on profile distributions under CCR.

**Figure 4:**
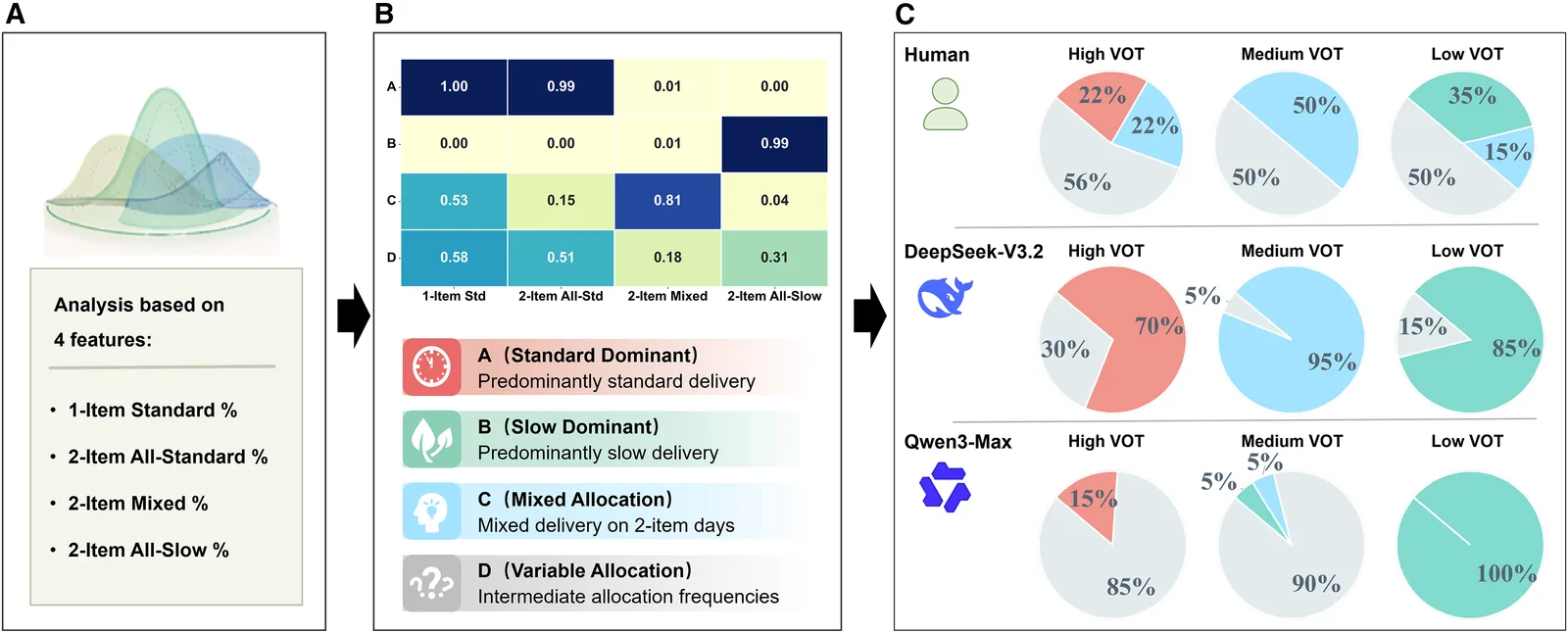
Identification and distribution of strategy portraits across participants under CCR (A) Feature selection for unsupervised clustering. Features comprise the standard delivery proportion on 1-item purchase days and the all-standard, mixed, and all-slow allocation proportions on 2-item purchase days. (B) Identification of strategy modes. Heatmap values represent the Gaussian mixture component means on the original 0–1 proportion scale. Modes A–D correspond to the standard dominant, slow dominant, mixed allocation, and variable allocation patterns, respectively. (C) Distributions of strategy portraits across participants under CCR. Pie charts show the percentages of eligible participant profiles assigned to each strategy mode within each participant-type-by-VOT group.

Four strategy modes were identified (modes A–D). Mode A (“standard dominant”) is characterized by a consistent tendency to choose standard delivery; mode B (“slow dominant”) exhibits a consistent tendency to choose slow delivery; mode C (“mixed allocation”) is distinguished by a high probability of choosing mixed delivery (one standard, one slow) in 2-item scenarios; and mode D (“variable allocation”) displays intermediate frequencies across the available delivery combinations.

Human participants exhibited multiple strategy modes within each VOT group under the CCR mechanism. In the BASE condition, the strategy portrait was primarily concentrated in mode A, with proportions ranging from 50% to 84% across VOT groups. Under CCR, the distribution of strategy modes became more dispersed. In the high-VOT group, the strategy portrait was distributed across mode A (22%), mode C (22%), and mode D (56%), with no single dominant mode. The medium-VOT portrait was evenly divided between modes C and D, with each accounting for 50%. The low-VOT portrait comprised modes B, C, and D, accounting for 35%, 15%, and 50%, respectively. Several allocation configurations, therefore, contributed to the human strategy portraits across all three VOT groups.

DeepSeek-v.3.2 strategy portraits changed systematically across the VOT groups. In the high-VOT group, mode A accounted for 70% of observations. In the medium-VOT group, mode C dominated with 95%. In the low-VOT group, mode B accounted for 85%. DeepSeek-v.3.2, therefore, showed a VOT-stratified transition from the standard dominant mode to the mixed allocation mode and then to the slow dominant mode.

Qwen3-Max, however, exhibited a different VOT-stratified strategy portrait. In the high-VOT group, mode D accounted for 85% of observations, while mode A accounted for 15%. In the medium-VOT group, mode D accounted for 90%, followed by mode B at 5% and mode C at 5%. All low-VOT observations were assigned to mode B. Only 24% of the high- and medium-VOT Qwen3-Max observations assigned to mode D retained the corresponding assignment after decision-state features were added.

Overall, the exploratory analysis described different VOT-stratified strategy mode compositions among the human, DeepSeek-v.3.2, and Qwen3-Max observations under the tested experimental configurations. Human observations were distributed across several strategy modes within the VOT groups. DeepSeek-v.3.2 observations followed a mode A-to-C-to-B pattern as VOT decreased, whereas Qwen3-Max observations were predominantly assigned to mode D at high and medium VOT and to mode B at low VOT. These findings characterize the strategy portraits generated by the samples and fixed LLM configurations examined in this study.

## Discussion

This study develops a controlled experimental framework to examine how humans and LLMs respond to a sequential reward-penalty incentive environment characterized by repeated decisions and intertemporal trade-offs. A key finding is that both humans and the tested LLMs respond substantially to the CCR mechanism, indicating that its incentive structure is sufficiently interpretable to elicit systematic choice adjustments. The similar pooled shifts in standard delivery proportions indicate that the tested LLM configurations captured the direction of the overall CCR response under the experimental composition examined, consistent with prior findings that LLMs can generate systematic responses in structured tasks.^41^^,^^42^^,^^43^

However, when considering policy responses, longitudinal response trajectories, and exploratory strategy portraits, a consistent pattern emerges: similar aggregate responses can coexist with divergent disaggregate behavioral patterns. In other words, aggregate correspondence alone does not extend uniformly to subgroup responses, temporal dynamics, or allocation-frequency profiles. This distinction is central to understanding both the promise and the limits of LLMs as behavioral proxies in sequential incentive policies.

From a dynamic perspective, the longitudinal results show three distinct response patterns. Humans exhibited gradual and sometimes incomplete adjustment. At high VOT, both their standard delivery proportion and BRR increased across rounds, which may reflect experience-based updating of task understanding and strategy. At medium VOT, however, the human standard delivery proportion remained close to the theoretical benchmark, while the BRR was substantially lower than that of DeepSeek-v.3.2. This contrast shows that aggregate benchmark proximity does not necessarily imply consistent trial-level best responses. DeepSeek-v.3.2 remained close to the VOT-specific benchmarks from the outset and maintained the highest overall BRR. This pattern may reflect a stable mapping from explicitly presented rules and state information to feedback-based one-step cost-minimizing choices. Qwen3-Max exhibited delayed adjustment, particularly at high and medium VOT, where both its standard delivery proportion and BRR increased markedly across rounds. This joint increase may reflect increasing sensitivity to the evolving feedback and decision states, although model-specific context processing and stochastic generation may also contribute. As a result, these model-specific trajectories ranged from early stabilization to delayed adjustment and should be interpreted as changes in conditional response generation, rather than learning directly comparable to human experience-based adjustment.

The exploratory strategy portraits reveal an additional layer beneath the aggregate results: aggregate-level correspondence did not imply that choices were organized similarly across the purchase contexts. Human portraits spanned multiple modes within each VOT group, whereas DeepSeek-v.3.2 showed VOT-ordered concentration in different dominant modes. Qwen3-Max was concentrated in mode D at high and medium VOT. The substantial reassignment of these mode D profiles after incorporating decision-state features indicates that frequency-based clustering may compress distinct state-conditioned response patterns. Together, these findings show that aggregate policy responses can obscure how choices are distributed across demand contexts, making conditional allocation structures an important complement to marginal adoption rates in LLM-based policy simulations.

Taken together, these findings provide a more nuanced understanding of the capabilities and limitations of LLM-based behavioral simulation. The tested LLM configurations generated systematic aggregate responses and, in several conditions, benchmark-proximate outcomes under the structured incentive rules, making them valuable tools for approximating aggregate behavioral responses under well-defined incentive structures. However, the VOT-stratified results, longitudinal results, and exploratory strategy portraits show that the aggregate correspondence does not necessarily extend to the disaggregate behavioral patterns. Thus, although LLMs may be useful for reproducing the direction of the aggregate policy response under the tested conditions, aggregate correspondence alone does not establish that they reproduce the processes generating human decisions.

From a broader perspective, these findings suggest that policy mechanisms involving cumulative incentives and delayed consequences may shape consumer decision-making in e-commerce logistics and related transportation contexts.^32^^,^^33^ At the same time, the observed differences between human and AI response patterns highlight that real-world policy effectiveness depends not only on incentive design but also on how individuals perceive, learn, and adapt to those incentives, underscoring the importance of incorporating behavioral frictions into policy design.

### Limitations of the study

Although this study provides insights into the differences in observable responses between humans and LLMs in the sequential reward-penalty incentive policy environments, several limitations remain. First, while the experimental design captures the core features of the CCR mechanism, it inevitably simplifies the complexities of real-world e-commerce logistics, failing to fully account for dynamic variables such as delivery cost variations and promotional discounts. Additionally, although the daily purchase demand (0, 1, or 2 items) reflects demand variability, it does not capture product heterogeneity (e.g., differences between daily necessities and urgent items) and struggles to replicate peak seasonal demand triggered by major shopping events such as “singles’ day” or 618. Future research could incorporate more realistic demand patterns and contextual variables to test the robustness of the findings. Second, due to experimental resource constraints, this study selected only DeepSeek-v.3.2 and Qwen3-Max as representative LLM agents. Because no formal prompt- or parameter-sensitivity analysis was conducted, the findings should be interpreted as specific to the tested configurations rather than as general properties of the broader model families (e.g., GPT series and Claude series). Future research should include additional models and systematically vary prompt design, history-window length, and decoding parameters to evaluate the robustness of LLM-based behavioral simulations. Finally, the humans in this study were primarily university students, and the sample was relatively limited, which may limit the generalizability of the conclusions to broader populations. Although existing research suggests that student samples are qualitatively similar to the general population in terms of behavioral patterns,^44^ quantitative differences may exist in the income levels, VOT distributions, and real-world delivery preferences. Future research should include more diverse samples encompassing different income levels, occupational backgrounds, and age groups to enhance the external validity of the findings.

## Resource availability

### Lead contact

Requests for further information and resources should be directed to and will be fulfilled by the lead contact, Zheng Zhu (zhuzheng89@zju.edu.cn).

### Materials availability

This study does not generate new unique materials.

### Data and code availability

The experiment data, the source code of the experimental platform, the scripts for human experiments (used for uploading to the platform to implement specific experiments), the scripts for LLM experiment, and the scripts for theoretical simulations, all have been made publicly available at Zenodo (Zenodo: https://doi.org/10.5281/zenodo.21903750). Any additional information required to reanalyze the data reported in this article is available from the lead contact upon request.

## Acknowledgments

This work was partially supported by the National Natural Science Foundation of China (72401255), Natural Science Foundation of Zhejiang province, China (LZ25E080007), “Pioneer” and “Leading Goose” R&D Program of Zhejiang, China (2024C012014), and the Smart Urban Future (SURF) Laboratory Zhejiang Province.

## Author contributions

Conceptualization, S.T. and Z.Z. methodology, S.T. and Z.Z.; platform development, testing, and optimization, Z.Z., S.T., and L.W.; experiments, S.T., Z.Z., and L.W.; writing – original draft, S.T. and Z.Z.; writing – review & editing, S.T., Z.Z., and L.W.; visualization, S.T. and Z.Z.; funding acquisition, Z.Z. and D.-H.L.

## Declaration of interests

The authors declare no competing interests.

## Declaration of generative AI and AI-assisted technologies in the manuscript preparation process

During the preparation of this work, the authors used GPT-5 in order to improve language and readability. After using this service, the authors reviewed and edited the content as needed and take full responsibility for the content of the publication article.

## STAR★Methods

### Key resources table

**Table:** 

REAGENT or RESOURCE	SOURCE	IDENTIFIER
**Software and algorithms**		

Python	Python Software Foundation	https://www.python.org
All code used in this paper	This paper	https://doi.org/10.5281/zenodo.21903750

### Experimental model and study participant details

For the human experiments, a total of 120 undergraduate and graduate students from Zhejiang University were recruited through the university’s internal website and randomly assigned to 10 independent 12-person groups, including five BASE groups and five CCR groups. The recruitment criteria were as follows: (1) at least 18 years old; (2) normal or corrected-to-normal vision; (3) good physical health, without major illnesses or interfering medications; (4) no history of mental or neurological disorders; and (5) ability to understand and comply with the study requirements. Following a post hoc data quality review, nine participants were excluded because their records contained repeated infeasible allocations, including positive standard delivery choices on multiple zero-demand days. These participants and their choices were removed before the system states and feedback trajectories used in the analyses were reconstructed. The final analytical sample comprised 111 participants (57 males and 54 females; mean age: 21.8 years in BASE and 22.6 years in CCR). The human experiment was administered in Chinese, and the complete task rules and settlement procedures were explained before the repeated decisions. All participants provided written informed consent before participation. The experimental procedures were approved by the Research Ethics Committee of the College of Biomedical Engineering & Instrument Science, Zhejiang University (approval reference: Zhejiang University Biomedical Engineering Ethics Review [2025] No. 4). Compensation consisted of a fixed base payment of 15 RMB and a performance-based incentive of 50–70 RMB determined by participants’ accumulated earnings over the six experimental rounds, resulting in a total payment of 65–85 RMB.

For the LLM experiments, we employed the model identifiers qwen3-max-2025-09-23 and deepseek-v3.2 through an OpenAI-compatible API. For each model and experimental condition, five independent 12-agent systems were simulated using the same experimental rules and prespecified purchase-demand sequences as the corresponding human systems. The human experiment was administered in Chinese, whereas the LLM prompts were presented in English. The complete BASE and CCR prompts are provided in Note S3. LLM prompt design. Temperature was set to 0.7, whereas top-p, maximum output tokens, and random seed were not explicitly specified and therefore followed the API provider’s default settings. Each agent operated independently and maintained a separate conversation history containing the system prompt and the three most recent complete daily interactions. Conversation histories continued across settlement rounds, whereas accumulated costs and carbon-credit balances were reset after each 20-day round. No fine-tuning or real-time parameter updating was performed. Final decisions were requested in the restricted numeric format {0}, {1}, or {2}, subject to the feasible range determined by the current demand. API-call failures were automatically retried up to five times. Invalid or infeasible final responses were excluded rather than recoded. One invalid DeepSeek-V3.2 decision was identified in the final data and excluded from the analysis.

### Method details

#### Validation of Statistical Analysis

To simulate the impact of order accumulation on logistics efficiency, the delivery time for standard delivery was modeled using a modified Bureau of Public Roads (BPR) function:

$$T_{s}\left(Q_{t}\right)=T_{0}\left(1+\alpha {\left(\frac{Q_{t}}{K}\right)}^{\beta }\right)$$

where *T*_*s*_(*Q*_*t*_) denotes delivery time as a function of daily order volume *Q*_*t*_, *T*_0_ is the free-flow delivery time, *K* represents system capacity, and *α*,*β* are scaling parameters.

In the experimental setting, the free-flow delivery time was set to *T*_0_ = 3 days, reflecting the baseline speed of standard delivery under uncongested conditions. The capacity parameter was specified as *K* = 6.8, with *α* = 0.15 and *β* = 2, consistent with commonly adopted parameterizations in transportation modeling. The daily volume of standard delivery orders was bounded within approximately 14–15, reflecting the upper range observed in the simulated environment. Substituting the upper bound into the function:

$$T_{s}\left(15\right)=3\left(1+0.15{\left(\frac{15}{6.8}\right)}^{2}\right)\approx 5.19$$

This result indicated that, even under high-demand conditions, the delivery time of standard logistics remained below the fixed lead time of slow delivery, which was set at 7 days. Therefore, despite congestion-induced delays, standard delivery retained a consistent time advantage within the decision environment.

#### Theoretical Equilibrium

To provide a theoretical benchmark for the experimental results, the equilibrium delivery strategies under the BASE and CCR were computed over a settlement period of 20 consecutive days. The purchase schedule was assumed to be exogenously determined before the optimization process. Therefore, the theoretical equilibrium was computed conditional on the realized purchase sequence and corresponded to an ex-post periodic Nash equilibrium.

Consider a settlement period consisting of *T* = 20 days. Let *i*=1,...,*N* denote consumers and *t*=1,...,*T* denote days. The realized purchase quantity of consumer *i* on day *t* is denoted by *q*_*it*_, where *q*_*it*_∈{0,1,2}. Consumer *i* decides the number of purchases assigned to the standard delivery service, denoted by *x*_*it*_, where *x*_*it*_∈*Z*, 0≤*x*_*it*_≤*q*_*it*_. The remaining *q*_*it*_-*x*_*it*_ purchases are assigned to the slow delivery service. Let *v*_*i*_ denote the value of time (VOT) of consumer *i*, and let $Q_{t}=\sum_{i=1}^{N}x_{it}$ denote the total standard delivery volume on day *t*. The delivery time of the standard delivery depends on the daily congestion level and is represented by the BPR function:

$$T_{s}\left(Q_{t}\right)=T_{0}\left(1+\alpha {\left(\frac{Q_{t}}{K}\right)}^{\beta }\right)$$

where *T*_0_ denotes the free-flow standard delivery time, *α*,*β* are scaling parameters, and *K* is the network capacity. The delivery time of the slow delivery is a constant denoted by *T*_*l*_.

Under the BASE, consumers minimize the total logistics cost accumulated over the entire settlement period:

$$C_{i}^{BASE}=\sum_{t=1}^{T}\left[x_{it}\left(v_{i}T_{s}\left(Q_{t}\right)+P_{s}\right)+\left(q_{it}-x_{it}\right)\left(v_{i}T_{l}+P_{l}\right)\right]$$

where *P*_*s*_ and *P*_*l*_ denote the prices of the standard and slow delivery, respectively. In this study, both delivery prices were set to zero.

Under the CCR, consumers additionally incur a terminal credit settlement at the end of each settlement period. The cumulative credit balance of consumer *i* is

$$B_{i}=r_{l}\sum_{t=1}^{T}\left(q_{it}-x_{it}\right)-r_{s}\sum_{t=1}^{T}x_{it}$$

where *r*_*s*_ and *r*_*l*_ denote the credit charge for standard delivery and the credit reward for slow delivery, respectively. The terminal settlement cost is defined as

$$S\left(B_{i}\right)=\left\{ \begin{array}{ll} -p_{r}B_{i}, & B_{i}\geq 0,\\ p_{c}\left(-B_{i}\right), & B_{i}<0, \end{array}\right.$$

where a positive credit balance is redeemed at *p*_*r*_ monetary units per credit and a negative balance is settled at *p*_*c*_ monetary units per credit. Accordingly, the consumer's objective function under CCR becomes

$${C_{i}^{CCR}=C}_{i}^{BASE}+S\left(B_{i}\right)$$

The theoretical benchmark is defined as a periodic Nash equilibrium. Let *x*_*i*_ denote the complete 20-day delivery strategy of consumer *i*, and let *x*_-*i*_ denote the strategy profile of all remaining consumers. An equilibrium strategy profile *x*∗ satisfies

$$C_{i}\left(x_{i}^{*},x_{-i}^{*}\right)\leq C_{i}\left(x_{i},x_{-i}^{*}\right),\forall i$$

indicating that no consumer can further reduce his or her total settlement-period cost through unilateral deviation while all other consumers keep their strategies unchanged.

The equilibrium was obtained using a sequential best response algorithm. Consumers sequentially updated their exact best responses until no profitable unilateral deviation existed. Convergence was declared when the maximum unilateral reduction in settlement-period cost was smaller than 10^-9^. To verify robustness, the equilibrium computation was repeated from three classes of initial strategy profiles, including all-standard delivery, all-slow delivery, and multiple random initializations. The equilibrium reported in this study was independent of the initial strategy profile at the aggregate level and therefore served as the theoretical benchmark for the subsequent experimental analysis.

#### Calculation of Best Response Rate

Building on the best response approach of Han et al. (2021),^45^ we calculated a best response rate (BRR) for each 20-day settlement round. For each positive-demand day, the participant’s feasible standard delivery allocation was *a*∈{0,…,*q*_*it*_}, where *q*_*it*_∈{1,2} denotes current demand. Days with zero demand were excluded because no delivery allocation was required.

For each feasible allocation, the one-step generalized cost was

$$c_{it}\left(a\right)=v_{i}\left[a\tau_{it}+\left(q_{it}-a\right)T_{l}\right]+S\left(b_{it}+6\left(q_{it}-a\right)-5a\right)-S\left(b_{it}\right)$$

where *v*_*i*_ denotes VOT, *τ*_*it*_ is the most recently observed standard delivery time, *T*_*l*_ = 7 days, and *b*_*it*_ is the carbon-credit balance before the decision.

The terminal settlement function was defined as

$$S\left(b\right)=\left\{ \begin{array}{ll} -2.1b, & b\geq 0\\ 4.2\left(-b\right), & b<0 \end{array}\right.$$

The published free-flow time of 3 days was used until standard delivery feedback became available. Consistent with the experimental information rule, *τ*_*it*_ was updated to the realized system delivery time only after the focal participant selected at least one standard delivery item. Credit balances were updated after each decision and reset after day 20 of each settlement round.

The myopic best response set was defined as $A_{it}^{*}=\arg\min_{a\in \left\{0,\ldots ,q_{it}\right\}}c_{it}\left(a\right)$. All cost-minimizing allocations within a numerical tolerance of 10^-9^ were retained in the case of ties. An observed allocation was coded as a best response when it belonged to $A_{it}^{*}$. Round-level BRR was calculated as

$${BRR}_{gr}=\frac{\sum_{\left(i,t\right)\in D_{gr}}1\left(x_{it}^{obs}\in A_{it}^{*}\right)}{\left|D_{gr}\right|}$$

where *D*_*gr*_ contains all eligible observed purchase-day decisions in system *g* and round *r*. When opponent allocations required to reconstruct system-level delivery time feedback were unavailable, they were completed using the corresponding theoretical equilibrium profile. Longitudinal BRR differences were estimated using binomial GEE, with the complete experimental system as the clustering unit. BRR therefore measures consistency with an information-set-conditioned, short-horizon optimum rather than full-horizon optimality.

#### Exploratory clustering

Decision-pattern profiles were constructed at the participant-within-system-and-condition level. Separate features were derived for 1-item and 2-item purchase days to represent the feasible allocation outcomes under each demand level. For 1-item days, the profile included the proportion of standard delivery choices. For 2-item days, the profile included the proportions of all-standard, mixed, and all-slow allocations. The clustering inputs comprised these four choice proportions; participant identity, VOT group, and experimental condition were used for post-clustering comparisons. BASE and CCR profiles were fitted jointly to define a common profile space.

The four proportion features were analyzed on their original 0–1 scale using a Gaussian mixture model with full covariance matrices. Candidate solutions with *K* = 2–7 were fitted using 10 random initializations and random seed 42 and were evaluated using AIC, BIC, silhouette coefficient, Calinski–Harabasz index, Davies–Bouldin index, maximum posterior classification probability, the proportion of uncertain assignments below 0.70, minimum class size, stability, parsimony, and correspondence between the component centers and distinguishable allocation configurations. Across the candidate solutions, AIC and BIC were minimized at *K* = 6. However, *K* = 4 was retained as a more parsimonious exploratory resolution because it provided four distinct and adequately sized strategy-level profiles with high posterior classification certainty and stable assignments across initialization and system-level resampling. The resulting profiles represented standard dominant, slow dominant, mixed allocation, and variable allocation patterns. Profile labels were assigned after estimation from the component means and were treated as descriptive summaries of observed allocation frequencies.

The four-component model had AIC = −7896.57, BIC = −7669.80, a silhouette coefficient of 0.501, a mean maximum posterior probability of 0.992, and 0.9% of profiles with a maximum posterior probability below 0.70. The smallest profile contained 39 participant-within-system-and-condition profiles. The component centers corresponded to a standard dominant profile (1-item standard = 0.996; 2-item all-standard = 0.989), a slow dominant profile (1-item standard = 0.005; 2-item all-slow = 0.986), a mixed allocation profile (1-item standard = 0.529; 2-item mixed = 0.809), and a variable allocation profile (1-item standard = 0.580; 2-item all-standard = 0.510, mixed = 0.181, and all-slow = 0.299). The respective profile sizes were 156, 47, 39, and 103.

Initialization stability was assessed across 100 single-start fits, producing a median ARI of 1.000 (2.5th–97.5th percentile, 0.989–1.000). Sampling stability was evaluated using 200 bootstrap resamples of complete experimental systems, stratified by Agent and condition, producing a median ARI of 0.809 (2.5th–97.5th percentile, 0.688–0.968). Sensitivity analyses examined the compositionally dependent all-slow feature, alternative covariance structures, alternative component numbers, and the addition of round, within-round position, reconstructed credit balance, and lagged-choice contrasts. Excluding the all-slow proportion yielded ARI = 0.811 relative to the four-feature solution. Alternative covariance structures yielded ARI values of 0.485–0.647. The state-augmented model yielded ARI = 0.424, with 63.6% of CCR assignments retained.

### Quantification and statistical analysis

Statistical analyses were performed in Python, with the complete experimental system treated as the independent unit. Q1–Q3 binary outcomes were analyzed using binomial GEEs with a working-independence correlation structure and bias-reduced system-clustered robust covariance; Q1 and Q2 used the prespecified stable period (rounds 5–6). Boundary theoretical benchmarks were summarized using system-level bootstrap intervals, and exact permutation or sign-flip tests provided small-sample sensitivity analyses. Q4 used an exploratory full-covariance Gaussian mixture model, with stability assessed across random initializations and system-level bootstrap resamples. All tests were two-sided, and Holm correction was applied within prespecified comparison families. Exact *p* values and 95% confidence intervals are reported where applicable; *p* < 0.05 was considered statistically significant.

## Supplemental information

Document S1. Figures S1 and S2 and Notes S1–S3

## References

[1] Xie Y., Liu S., Chen H., Cao S., Zhang H., Feng D., Wan Q., Zhu J., Zhu Q., Zhu Q. (2025). Localization, balance, and affinity: A stronger multifaceted collaborative salient object detector in remote sensing images. IEEE Trans. Geosci. Remote Sens..

[2] Xie Y., Zhan N., Zhu J., Xu B., Chen H., Mao W., Luo X., Hu Y., Hu Y. (2024). Landslide extraction from aerial imagery considering context association characteristics. Int. J. Appl. Earth Obs. Geoinf..

[3] Song W., Cui X., Xie Y., Wang G., Ma J. (2025). Monocular depth estimation via a detail semantic collaborative network for indoor scenes. Sci. Rep..

[4] Song W., Zhao Y., Tu J., Chen M., Xie Y., Cui X. (2026). A visual attention-guided approach for concrete crack detection in complex environments. Eng. Appl. Artif. Intell..

[5] Song W., Chen M., Zhao Y., Xie Y., Song C., Huo J., Zheng C., Cui X., Cui X. (2026). SDANet: A structure-detail aware network for image restoration in enclosed building spaces under fire-smoke conditions. Dev. Built Environ..

[6] Wei J., Tay Y., Bommasani R., Raffel C., Zoph B., Borgeaud S., Fedus W. (2022). Emergent abilities of large language models. arXiv.

[7] Bubeck S., Chandrasekaran V., Eldan R., Gehrke J., Horvitz E., Kamar E., Zhang Y. (2023). Sparks of artificial general intelligence: Early experiments with GPT-4. arXiv.

[8] Schoenegger P., Tuminauskaite I., Park P.S., Bastos R.V.S., Tetlock P.E. (2024). Wisdom of the silicon crowd: LLM ensemble prediction capabilities rival human crowd accuracy. Sci. Adv..

[9] Park J.S., O'Brien J., Cai C.J., Morris M.R., Liang P., Bernstein M.S. (2023). Proceedings of the 36th annual acm symposium on user interface software and technology.

[10] Levitt S.D., List J.A. (2007). What do laboratory experiments measuring social preferences reveal about the real world?. J. Econ. Perspect..

[11] Fontana N., Pierri F., Aiello L.M. (2025). Nicer than humans: how do large language models behave in the prisoner's dilemma?. Proceedings of the International AAAI Conference on Web and Social Media.

[12] Jiang G., Yang S., Wang Y., Hui P. (2026). When trust collides: Exploring human-LLM cooperation intention through the prisoner’s dilemma. Int. J. Hum. Comput. Stud..

[13] Guo F. (2023). GPT in game theory experiments. arXiv.

[14] Ragavi O.E., Ashok P. (2025). LLMs are not weird: Comparing AI and human financial decision-making. J. Behav. Exp. Econ..

[15] Wang L., Jiang Z., Hu C., Zhao J., Zhu Z., Chen X., Wang Z., Liu T., He G., Yin Y. (2025). Comparing AI and human decision-making mechanisms in daily collaborative experiments. iScience.

[16] Xu Y., Wang S., Li P., Luo F., Wang X., Liu W., Liu Y. (2023). Exploring large language models for communication games: An empirical study on werewolf. arXiv.

[17] Bakhtin A., Brown N., Dinan E., Farina G., Flaherty C., Fried D., Goff A., Gray J., Hu H., Meta Fundamental AI Research Diplomacy Team FAIR† (2022). Human-level play in the game of diplomacy by combining language models with strategic reasoning. Science.

[18] Binz M., Schulz E. (2023). Using cognitive psychology to understand GPT-3. Proc. Natl. Acad. Sci. USA.

[19] Xiao F., Wang X.T.X. (2025). Evaluating the ability of large Language models to predict human social decisions. Sci. Rep..

[20] Ashery A.F., Aiello L.M., Baronchelli A. (2025). Emergent social conventions and collective bias in LLM populations. Sci. Adv..

[21] Capraro V. (2026). LLMorphism: When humans come to see themselves as language models. arXiv.

[22] Loru E., Nudo J., Di Marco N., Santirocchi A., Atzeni R., Cinelli M., Cestari V., Rossi-Arnaud C., Quattrociocchi W., Quattrociocchi W. (2025). The simulation of judgment in LLMs. Proc. Natl. Acad. Sci. USA.

[23] Quattrociocchi W., Capraro V., Perc M. (2025). Epistemological fault lines between human and artificial intelligence. arXiv.

[24] Quattrociocchi W., Capraro V., Marcus G. (2026). Statistical approximation is not general intelligence. Nature.

[25] Grand View Research (2020).

[26] Jaller M., Pahwa A. (2020). Evaluating the environmental impacts of online shopping: A behavioral and transportation approach. Transport. Res. Transport Environ..

[27] Guo X., Lujan Jaramillo Y.J., Bloemhof-Ruwaard J., Claassen G.D.H. (2019). On integrating crowdsourced delivery in last-mile logistics: A simulation study to quantify its feasibility. J. Clean. Prod..

[28] Buldeo Rai H., Broekaert C., Verlinde S., Macharis C. (2021). Sharing is caring: How non-financial incentives drive sustainable e-commerce delivery. Transport. Res. Transport Environ..

[29] Kokkinou A., Quak H., Mitas O., Mandemakers A. (2024). Should I wait or should I go? Encouraging customers to make the more sustainable delivery choice. Res. Transport. Econ..

[30] Liu X., Zhou Z., Yuen K.F., Wang X. (2024). Green and gamified! An investigation of consumer participation in green last-mile from a gamification affordance perspective. J. Retailing Consum. Serv..

[31] Waters M. (2020).

[32] Ding H., Yang H., Qin X., Xu H. (2023). Credit charge-cum-reward scheme for green multi-modal mobility. Transp. Res. Part B Methodol..

[33] Hu C., Wang K., Wang L., Zhao J., Zhu Z., Yang H., Ding H., He G., Wang Z., Wang Z. (2025). Downward comparisons enhance carbon credit policy sustainability: Lab evidence. iScience.

[34] Small K.A. (2012). Valuation of travel time. Economics of Transportation.

[35] Oyama Y., Fukuda D., Imura N., Nishinari K. (2024). Do people really want fast and precisely scheduled delivery? E-commerce customers' valuations of home delivery timing. J. Retailing Consum. Serv..

[36] Simon H.A. (1955). A behavioral model of rational choice. Q. J. Econ..

[37] Prystawski B., Mohnert F., Tošić M., Lieder F. (2022). Resource-rational models of human goal pursuit. Top. Cogn. Sci..

[38] Sweller J. (1988). Cognitive load during problem solving: Effects on learning. Cogn. Sci..

[39] Paas F., Renkl A., Sweller J. (2003). Cognitive load theory and instructional design: Recent developments. Educ. Psychol..

[40] Dunovan K., Verstynen T. (2019). Errors in action timing and inhibition facilitate learning by tuning distinct mechanisms in the underlying decision process. J. Neurosci..

[41] Webb T., Holyoak K.J., Lu H. (2023). Emergent analogical reasoning in large language models. Nat. Hum. Behav..

[42] Lampinen A., Dasgupta I., Chan S., Mathewson K., Tessler M., Creswell A., Hill F. (2022). Findings of the Association for Computational Linguistics: EMNLP 2022.

[43] Mitchell M., Krakauer D.C. (2023). The debate over understanding in AI’s large language models. Proc. Natl. Acad. Sci. USA.

[44] Belot M., Duch R., Miller L. (2015). A comprehensive comparison of students and non-students in classic experimental games. J. Econ. Behav. Organ..

[45] Han X., Yu Y., Jia B., Gao Z.-Y., Jiang R., Zhang H.M. (2021). Coordination behavior in mode choice: Laboratory study of equilibrium transformation and selection. Prod. Oper. Manag..

