# Supplementary material for: Similar aggregate but divergent disaggregate responses in human and AI under sequential reward-penalty incentives
Source: iScience. 2026 Oct 6;29(11):117800. doi: 10.1016/j.isci.2026.117800 (PMC13676151; doi:10.1016/j.isci.2026.117800)
Supplement: Supplementary file 1 [file mmc1.pdf]

iScience, Volume *29*

Supplemental information

Similar aggregate but divergent disaggregate responses in human and AI under sequential reward-penalty incentives

Sihan Tao, Linghao Wang, Zheng Zhu, and Der-Horng Lee

Supplementary Information for

Similar Aggregate Responses but Divergent Disaggregate Behavioral Patterns: Human and AI under Sequential Reward-Penalty Incentives

Sihan Tao[1], Linghao Wang[2], Zheng Zhu[2,4,*], Der-Horng Lee[3,*]

1 Institute of Intelligent Transportation Systems & Polytechnic Institute, Zhejiang University, Hangzhou, China

2 Institute of Intelligent Transportation Systems, College of Civil Engineering and Architecture, Zhejiang University, Hangzhou, China

3 Zhejiang University–University of Illinois Urbana Champaign Institute, Zhejiang University, Hangzhou, China

4 Lead contact

* Corresponding author, email: zhuzheng89@zju.edu.cn, dhlee@intl.zju.edu.cn

This Supplementary Information includes:

Supplementary Notes: 1 to 3

Supplementary Figures: 1 to 2

Supplementary Notes

Note S1. Informed Consent Form

Party A: Participant

Party B: Organizing team

Dear Sir/Madam,

You are invited to participate in a scientific research experiment titled “Experiments on Multi-Participant Collaborative Decisions under a Policy in E-commerce Logistics” This experiment is funded by the National Natural Science Foundation of China. Before deciding whether to participate, please carefully read the following information. It will help you understand the experiment's benefits, risks, responsibilities, and rights.

1. Benefits of Party A

Participation in this experiment will provide deeper insights into the delivery decision-making processes. The findings may assist in improving your future delivery choices.

Participants who complete the experiment will receive a monetary reward ranging from 65 to 85 RMB, based on their performance.

2. Potential Risks of Party A

This experiment adheres strictly to established protocols. You will only be required to complete online tasks. If you experience any discomfort or wish to withdraw from the study at any time, please inform the staff immediately, and assistance will be provided.

3. Responsibility Party A

Participants are required to arrive at the laboratory on time and must not leave without permission. During the experiment, participants must follow the instructions provided by the experimenters, complete the tasks diligently, and refrain from engaging in unsolicited communication with others.

4. Responsibility of Party B

In the event of any unforeseen incidents during the experiment, appropriate medical costs will be covered by Party B.

All information related to your participation in this experiment will be stored on a local server. Experimental results, including personal data, will remain strictly confidential

within the limits of applicable laws. Your name will not appear in any reports; instead, only the unique ID assigned to you during the experiment will be used.

5. Rights of Party B

Party B has the right to use and analyze the data from the experiment and publish in related academic journals without disclosing your personal information.

Consent Declaration

Party A

1. I have read this informed consent form and fully understand the benefits, risks, responsibilities, and rights associated with this experiment.

2. I have had sufficient time to make my decision.

3. I voluntarily agree to participate in the scientific research described in this form and consent to the use of my data for publication purposes.

4. I consent to the review of my research records by representatives of the Ethics Committee or funding agencies as required.

5. I will receive a signed and dated copy of this informed consent form.

Finally, I agree to participate in this experiment.

Participant Signature: Date: ______ Year ______ Month ______ Day

Participant Contact Information: ________________________

Party B

I confirm that I have explained the details about the benefits, risks, responsibilities and rights of this experiment to the participant. I have also provided the participant with a signed copy of the informed consent form.

Researcher Signature: Date: ______ Year ______ Month ______

Researcher Contact Information: ________________________

Note S2. E-commerce Logistics Delivery Choice Experiment Instructions

BASE Experiment

Background

This research focuses on consumers' logistics delivery choice behavior in e-commerce settings and its implications for sustainable logistics systems. The rapid growth of e-commerce has led to increasing environmental concerns, particularly in last-mile delivery. Understanding how consumers make delivery choices is therefore critical for optimizing logistics systems. This experiment, conducted in an e-commerce context, aims to investigate how consumers with different characteristics make delivery decisions.

Instructions

Before the experiment: you will be assigned a unique ID and password to log into a designated computer in the lab. The experimental setup and key terminologies will be explained by the organizers.

During the experiment: you will answer questions within the experimental system. The number of experimental rounds and the tasks in each round will be specified at the beginning. After completing each task, you will wait for others to finish before proceeding to the next round. At the end of each round, scores will be assigned to each participant. Completion of all rounds signifies the conclusion of the experiment.

After the experiment: you will receive monetary reward based on your total score.

Experimental procedure

Participants, all aged 18 or older, will engage in group experiments of 12 individuals each. During the experiment, participants will answer scenario-based questions displayed on a computer. Each experiment will last between 40 minutes and 1 hour, simulating repeated delivery decision-making in a multi-day e-commerce context.

As shown in **Figure 1**, participants are tasked with making delivery mode choices each day. They will decide between standard delivery (Free, 3+ days) and slow delivery (Free, 7 days).

Participants will be divided into three groups based on their Value of Time (VOT): high (18 RMB/day), medium (10 RMB/day), and low (3 RMB/day). VOT refers to the monetary amount a participant is willing to pay to save one unit of delivery time. The objective of the experiment is to minimize total delivery costs. On each virtual decision day, participants make delivery choices for 0, 1, or 2 items, selecting how many items

to send via standard delivery, with the remaining items automatically assigned to slow delivery. After each choice, the number of times choosing the standard delivery will determine the delivery time. The simulation runs for 20 days (rounds), with the interface displaying and participants making their decisions via mouse clicks during each round.

During the experiment, participants are provided with delivery information. This information includes total rounds, current round, current day, assigned VOT group, number of participants, number of items bought today, last standard delivery time, yesterday's delivery cost, and current round's cumulative cost, which participants used to select their delivery method for the next interval.

The final experimental compensation consisted of two components: a base reward and a performance-based incentive. The base reward is fixed at 15 RMB, while the incentive reward is fluctuated between 50 to 70 RMB, depending on how well participants manage delivery times and carbon credits. The total reward is based on the accumulation of earnings over six rounds, which will be converted into RMB according to the participant's VOT group. For the high VOT group, the initial token is 2350, and the conversion ratio is 100:4.5; for the medium VOT group, the initial token is 1370, with a conversion ratio of 100:5.5; and for the low VOT group, the initial token is 500, with a conversion ratio of 100:7. This ensures that, although the token amounts and conversion rates differ, the average compensation across all groups remains approximately equal, ranging from 65 to 85 RMB.

CCR Experiment

Background

This research focuses on consumers' logistics delivery choice behavior in e-commerce settings and its implications for sustainable logistics systems. The rapid growth of e-commerce has led to increasing environmental concerns, particularly in last-mile delivery. While traditional views emphasize speed, recent studies suggest that consumers are gradually shifting from a single preference for speed toward a more comprehensive consideration of cost, reliability, and environmental impact. Understanding how consumers make delivery choices under such trade-offs is therefore critical for optimizing logistics systems. The Credit-Charge-cum-Reward (CCR) mechanism is an incentive-based approach that encourages more sustainable choices

through cumulative rewards and penalties. This experiment, conducted in an e-commerce context, aims to investigate how consumers with different characteristics make delivery decisions under CCR.

Instructions

Before the experiment: you will be assigned a unique ID and password to log into a designated computer in the lab. The experimental setup and key terminologies will be explained by the organizers.

During the experiment: you will answer questions within the experimental system. The number of experimental rounds and the tasks in each round will be specified at the beginning. After completing each task, you will wait for others to finish before proceeding to the next round. At the end of each round, scores will be assigned to each participant. Completion of all rounds signifies the conclusion of the experiment.

After the experiment: you will receive monetary reward based on your total score.

Experimental procedure

Participants, all aged 18 or older, will engage in group experiments of 12 individuals each. During the experiment, participants will answer scenario-based questions displayed on a computer. Each experiment will last between 40 minutes and 1 hour, simulating repeated delivery decision-making in a multi-day e-commerce context.

As shown in **Figure 2**, participants are tasked with making delivery mode choices each day. They will decide between standard delivery (Free, 3+ days, -5 credits) and slow delivery (Free, 7 days, +6 credits). During settlement, if the balance is positive, it will be converted into a bonus at a rate of 2.1 RMB per point; if negative, a penalty will be paid at a rate of 4.2 RMB per point.

Participants will be divided into three groups based on their Value of Time (VOT): high (18 RMB/day), medium (10 RMB/day), and low (3 RMB/day). VOT refers to the monetary amount a participant is willing to pay to save one unit of delivery time. The objective of the experiment is to minimize total costs, including delivery costs and carbon credit costs. Each participant's carbon credit account starts with a balance of zero. On each virtual decision day, participants make delivery choices for 0, 1, or 2 items, selecting how many items to send via standard delivery, with the remaining items automatically assigned to slow delivery. After each choice, the number of times

choosing the standard delivery will determine the delivery time. The simulation runs for 20 days (rounds), with the interface displaying and participants making their decisions via mouse clicks during each round.

During the experiment, participants are provided with delivery information. This information includes total rounds, current round, current day, assigned VOT group, number of participants, number of items bought today, last standard delivery time, yesterday's delivery cost, current round’s cumulative cost and carbon credits balance, which participants used to select their delivery method for the next interval.

The final experimental compensation consisted of two components: a base reward and a performance-based incentive. The base reward is fixed at 15 RMB, while the incentive reward is fluctuated between 50 to 70 RMB, depending on how well participants manage delivery times and carbon credits. The total reward is based on the accumulation of earnings over six rounds, which will be converted into RMB according to the participant’s VOT group. For the high VOT group, the initial token is 2360, and the conversion ratio is 100:4.5; for the medium VOT group, the initial token is 1450, with a conversion ratio of 100:5.5; and for the low VOT group, the initial token is 380, with a conversion ratio of 100:7. This ensures that, although the token amounts and conversion rates differ, the average compensation across all groups remains approximately equal, ranging from 65 to 85 RMB.

Note S3. LLM Prompt Design

We employ a multi-round iterative method for the design of prompts to ensure the smooth progression of the game. The game descriptions are crafted based on experimental contexts. The structure of these prompts comprises four fundamental elements: game rules, user tasks, a template for announcing game outcomes (used for displaying previous results), and specifications for format responses. The specific design is as follows:

BASE Experiment

SYSTEM You are a participant in an e-commerce logistics decision experiment. Your ID is {userid}. You belong to the {group} with a value of time (VOT) of {vot} RMB/day. VOT refers to the monetary value that consumers are willing to pay to save on the unit delivery time, reflecting their preferences and willingness to pay regarding delivery speed.

Your goal:

Minimize your final total cost for each round.

The mechanism:

The experiment consists of six rounds. Each round lasts {cycle_length} days. At the end of each round, the accumulated cost will reset. Every day, you will purchase a certain number of items (0, 1, or 2). You only need to decide how many items to send via standard delivery. The remaining items will automatically be sent via slow delivery. Formula: Slow_Count = Total_Items - Standard_Count.

Rules:

1. Slow Delivery: Takes 7 days fixed. Cost = Items * VOT * 7.

2. Standard Delivery: Takes roughly 3 days, but increases if many people use it (congestion). Cost = Items * VOT * Actual_Time.

3. Constraint: You cannot choose standard delivery more times than the items you bought. (e.g., if you bought 1 item, choice must be {{0}} or {{1}}).

Before making a decision, you will be provided with last standard delivery time, yesterday's cost and current cycle accumulated cost from the previous day:

You must output your choice strictly in this format: {{0}} or {{1}} or {{2}}.

USER Day: {step}. Current Cycle Round: {round}. Items Bought Today: {items}. Last Standard Delivery Time: {standard_time}. Yesterday's Cost (Pure Logistic): {cost_yesterday}. Current Cycle Accumulated Cost: {total_cost}. Please analyze the current situation based on the information above.

ASSISTANT Let’s analyze.....

USER Based on your analysis above, please make your final decision. You bought {items} items today. How many items do you want to send via standard delivery? Strictly answer with format {{0}}, {{1}} or {{2}}.

ASSISTANT {{0}}

CCR Experiment

SYSTEM You are a participant in an e-commerce logistics decision experiment with a credit charge-cum-reward mechanism. Your ID is {userid}. You belong to the {group} with a value of time (VOT) of {vot} RMB/day. VOT refers to the monetary value that consumers are willing to pay to save on the unit delivery time, reflecting their preferences and willingness to pay regarding delivery speed.

Your goal:

Minimize your final total cost for each round. Formula: Final Total Cost = Cycle Accumulated Cost (Pure Logistic) - Carbon Credit Cost. Note: A positive credit value reduces your cost (bonus), while a negative value increases it (penalty).

The mechanism:

The experiment consists of six rounds. Each round lasts {cycle_length} days. At the end of each round, the accumulated cost and carbon credit balance will reset. Every day, you will purchase a certain number of items (0, 1, or 2). You only need to decide how many items to send via standard delivery. The remaining items will automatically be sent via slow delivery. Formula: Slow_Count = Total_Items - Standard_Count.

Credit charge-cum-reward rules:

1. Account:

- Your account starts at 0 every round. Points accumulate daily.

2. Standard Delivery:

- Time: ~3 days (dynamic, increases if many people use it).

- Logistic Cost: Items * VOT * Actual_Time.

- Carbon Points: deduct 5 points per item (-5).

3. Slow Delivery:

- Time: Fixed 7 days.

- Logistic Cost: Items * VOT * 7.

- Carbon Points: add 6 points per item (+6).

4. Settlement:

- Credits are not converted to money daily. They are only settled at the end of the 20-day round.

- At day 20 (cycle end), your total credits are converted as follows:

[A] If total credits > 0 (positive): You earn 2.1 RMB per point (reward).

[B] If total credits < 0 (negative): You pay 4.2 RMB per point (penalty).

- After settlement, credits are reset to 0 for the next round.

5. Constraint: You cannot choose standard delivery more times than the items you bought. (e.g., if you bought 1 item, choice must be {{0}} or {{1}}).

Before making a decision, you will be provided with last standard delivery time, yesterday's cost (pure logistic), carbon credit balance and current cycle accumulated cost (pure logistic) from the previous day. You must output your choice strictly in this format: {{0}} or {{1}} or {{2}}.

USER Day: {step}. Current Cycle Round: {round}. Items Bought Today: {items}. Last Standard Delivery Time: {standard_time}. Yesterday's Cost (Pure Logistic): {cost_yesterday}. Carbon Credit Balance: {balance}. Current Cycle Accumulated Cost (Pure Logistic): {total_cost}. Please analyze the current situation based on the information above.

ASSISTANT Let’s analyze......

USER Based on your analysis above, please make your final decision. You bought {items} items today. How many items do you want to send via standard delivery? Strictly answer with format {{0}}, {{1}} or {{2}}.

ASSISTANT {{0}}

Supplementary Figures

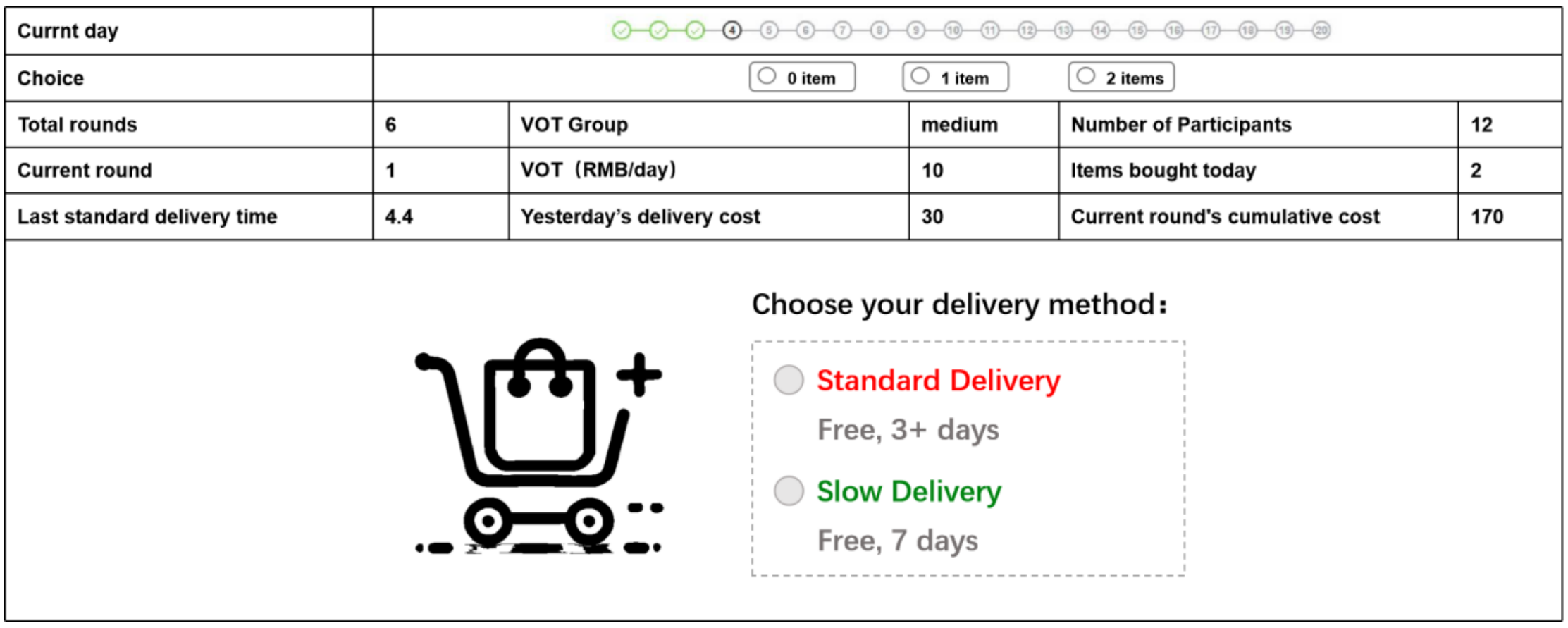

Supplementary Figure 1. BASE experimental interface

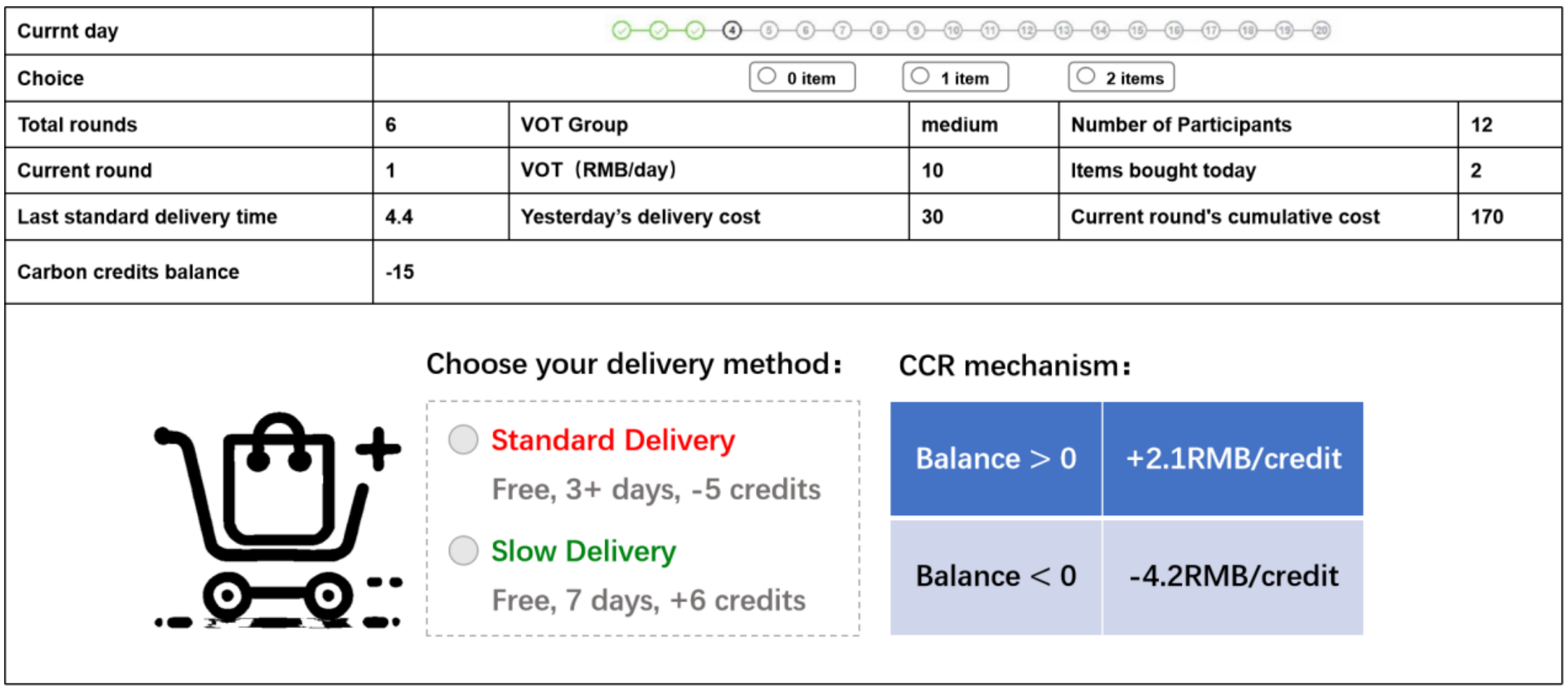

Supplementary Figure 2. CCR experimental interface
